# CONSORT: missing missing data guidelines, the effects on HTA monograph reporting

**DOI:** 10.1186/1745-6215-12-S1-A61

**Published:** 2011-12-13

**Authors:** Yvonne Sylvestre

## Objectives

The reporting of randomised controlled trials (RCTs) has improved since the publication of the first CONSORT statement. However, word limitations on journal articles still preclude full description of methodology. The extended format of HTA monographs enable trialists to fully articulate methods. We assess the impact of the lack of reference to missing data in CONSORT on the reporting of RCTs in HTA monographs and compare with randomisation, blinding, and allocation concealment which are included.

## Methods

The methodology for the comparison for the randomisation, blinding and allocation concealment followed the structure used by Hopewell et al. when assessing the impact of the updates of CONSORT. Detail of these on methodological CONSORT items were extracted for each monograph identified using a 1996 and 2001 CONSORT item check list for the periods 1997-2001, 2002-2005 and 2006-2010 respectively. Screening and data extraction was carried out independently by two authors determining whether each item was reported. Any discrepancies were referred to the other authors and resolved through discussion and consensus. We extended this methodology for whether missing data occurred and was described, and whether imputation and a sensitivity analysis were used. Additionally the methods of treatment of missing data were recorded.

## Results

We identified 517 monographs which yielded 93 parallel group, crossover, cluster, factorial and cost-effectiveness RCTs for assessment. Across the three periods randomisation reporting increased from 70% to 92%, allocation concealment from 70% to 90% while blinding varied between 70% and 83%. Missing data issues were rarely discussed in a missing data section. Some details could be identified by careful reading of methods section, however the details of imputation methods used were only rose from 40% to 71%. The details of a sensitivity analysis are only reported in 30% of studies.

## Conclusions

Most topics examined were more frequently reported in recent years, however missing values information is still not present in one quarter of RCT reports, and is more poorly reported than items specifically listed in CONSORT. We often experienced difficulty in establishing which methodology was used and whether the statistical and health economics analyses used the same methods. We recommend that missing data be included within the CONSORT checklist to improve the reporting of an important element of trial analysis.

